# Detecting glioblastoma infiltration beyond conventional imaging tumour margins using MTE-NODDI

**DOI:** 10.1162/imag_a_00472

**Published:** 2025-02-18

**Authors:** Saketh R. Karamched, Dunja Gorup, Daniele Tolomeo, Lucy J. Brooks, Ting Gong, Andreas Christ Sølvsten Jørgensen, Ciaran Scott Hill, Melanie Clements, Tammy L. Kalber, Jack A. Wells, Daniel J. Stuckey, Lewis Thorne, Vahid Shahrezaei, Samuel Marguerat, Hui Zhang, Simona Parrinello, Mark F. Lythgoe

**Affiliations:** UCL Centre for Advanced Biomedical Imaging, Division of Medicine, University College London, London, United Kingdom; Samantha Dickson Brain Cancer Unit, UCL Cancer Institute, London, United Kingdom; IXICO plc, London, United Kingdom; UCL Hawkes Institute, Department of Computer Science, University College London, London, United Kingdom; Department of Mathematics, Faculty of Natural Sciences, Imperial College London, London, United Kingdom; I-X Centre for AI In Science, Imperial College London, London, United Kingdom; Department of Neurosurgery, The National Hospital for Neurology and Neurosurgery, London, United Kingdom; UCL Cancer Institute, London, United Kingdom

**Keywords:** glioblastoma, tumour cells, tumour invasion, diffusion-weighted imaging, NODDI, multiple echo-time NODDI, imaging biomarkers, translational imaging

## Abstract

Glioblastoma (GBM) is the most common and aggressive brain tumour with starkresistance to available therapies, leading to relapse and a median survival of<15 months. A key cause of therapy resistance is diffuse infiltration oftumour cells into brain regions surrounding the tumour, which presents a majorclinical challenge as existing imaging techniques offer limited detection of theresectable margin. Here, we use diffusion weighted imaging (DWI) and apply themultiple echo time neurite orientation dispersion and density imaging(MTE-NODDI) model as a tool to detect tumour cells in the hard-to-distinguishmargin. We used the G144 patient-derived xenograft model, with characteristicinvasion along white matter tracts, in combination with MTE-NODDI. Tumourdevelopment was monitored, and magnetic resonance imaging (MRI) data wereacquired over a 4-week period, starting at 4 weeks after stereotactic injectionof tumour cells. MTE-NODDI demonstrated sensitivity to the developing tumour inthe invading margin, and changes in measured parameters were apparent from 6weeks after injection. In comparison to standard DWI, MTE-NODDI showed increasedsensitivity to the tumour-associated changes in the margin. Furthermore,extraneurite volume fraction (*f_en_*) and neuritedensity index (NDI) measured from MTE-NODDI correlated with immunohistologicalmeasurement of tumour cells. These findings suggest that MTE-NODDI maynon-invasively detect infiltrating cells and tumour-induced pathology in marginregions without T2 or DWI changes in a patient-derived mouse model of GBM.MTE-NODDI is clinically translatable and could be a powerful tool forneurosurgeons to maximise surgical resection, resulting in better survivaloutcomes for patients with GBM.

## 1 Introduction

Glioblastoma (GBM) is the most common and aggressive primary brain tumour in adults(Omuro & DeAngelis,2013). The current standard of care includes maximal safe surgicalresection followed by radiotherapy and concomitant chemotherapy with temozolomide(Stupp et al., 2005).Despite such active treatment strategies, the prognosis remains extremely poor andthe outcome for nearly all patients is dismal, with a median survival of <15months and an average 5-year survival rate of <5% (Miller et al., 2021). The keycause of resistance to available treatments is diffuse infiltration of tumour cellsinto the normal brain parenchyma, a hallmark of GBM (Cuddapah et al., 2014;Vehlow & Cordes, 2013). Infiltration precludescurative surgery and causes recurrence from low numbers of cells that have invadedpast the margin of original resection. Radiotherapy target volumes are planned toaccount for an unseen population of invading tumour cells into healthy tissues thatcan extend beyond the gross tumour volume (Niyazi et al., 2023), yet tumour recurrence occurs,possibly indicating migrating tumour cells outside the planned treatment area. Ascell invasion may not be uniform from the tumour bulk, an image-guided approach mayprovide a method to more closely map migrating tumour cells and thus allowing a moreprecisely tailored therapy plan. Invasion in GBM is thus a major clinical challengebecause traditional imaging methods, which include post-gadolinium contrast-enhancedT1-weighted, T2-weighted and fluid attenuated inversion recovery (FLAIR) MRI, cannotdetect these infiltrating cells, making it impossible to accurately determine tumourmargins (Lasocki & Gaillard,2019;Price &Gillard, 2011). Other methods to aid tumour delineation include5‑aminolevulinic acid (5‑ALA)-guided surgery as an adjunct to maximiseresection (Hadjipanayis et al.,2015;Schatlo et al.,2015;Stepp &Stummer, 2018;Stummer etal., 2006) and intraoperative MRI (Kubben et al., 2011;Schatlo et al., 2015). However, these imaging techniquesoffer limited identification of the invading margin, which inevitably leads totumour recurrence within 2 cm of the original tumour site in 80–90% of cases(Chamberlain, 2011;Claes et al., 2007;De Bonis et al., 2013;Petrecca et al., 2013;Vehlow & Cordes, 2013).Recent studies utilising magnetic resonance spectroscopy (MRS) (Laack et al., 2021),^18^F-DOPA positron emission tomography (PET) (Laack et al., 2021), and advanced MRI (Kim et al., 2021) fordose-escalated radiation therapy have shown early promise, suggesting that improvingtumour volume definition can lead to better treatment planning in GBM.

Diffusion weighted imaging (DWI) is sensitive to diffusion of water molecules inbiological tissues. DWI techniques provide non-invasive indices associated with thepattern of water diffusion, which reflect tissue microstructure (Basser et al., 1994;Le Bihan, 2014). As GBM cellsinfiltrate away from the tumour bulk into adjacent brain tissues, they confront newand heterogenous microenvironments, resulting in tumour-affected changes to theunderlying tissue. As such, DWI may provide improved sensitivity to microstructuralchanges caused by the invading tumour, in turn delineating margins outside thetraditionally identified tumour bulk (Maier et al., 2010;Sugahara et al., 1999;Thoeny & Ross, 2010). Neurite orientation dispersion and densityimaging (NODDI) is an advanced method of DWI, which uses a biophysicalcompartment-based model for studying microstructural changes of brain tissue (Zhang et al., 2012). NODDI assumesthat the signal measured from each tissue voxel originates from a combination ofthree types of microstructural compartments: intra-neurite, extra-neurite, and freewater volume fractions, each of which have a unique effect on water diffusion withinthe environment. This allows for compartment-specific markers of microstructuralproperties to be determined, such as the neurite density index (NDI) (the fractionof tissue that comprises neurites; axons or dendrites), the extra-neurite volumefraction (*f_en_*) (the fraction of tissue other than theneurites including microglia, astrocytes, oligodendrocytes, soma, ependymal cells,extra-cellular matrices, and vasculature), and orientation dispersion index (ODI),which reflects the spatial configuration of the neurite structures (Zhang et al., 2012). NODDI-derivedmeasures are sensitive to altered tissue microstructure in brain development,maturation, and aging (Cox et al.,2016;Kamiya et al.,2020), as well as numerous neuropathological conditions (Churchill et al., 2019;Colgan et al., 2016;Fu et al., 2020;Kamagata et al., 2017;Kamiya et al., 2020;Kraguljac et al., 2021;Mastropietro et al., 2019;Mitchell et al., 2019;Nazeri et al., 2017;Palacios et al., 2020;Parker et al., 2018;Schneider et al., 2017;Sone et al., 2018;Spano et al., 2018;Z. Wang et al., 2019;Winston et al., 2014). Previousstudies have reported the application of NODDI in investigating brain tumour bulk,differentiating primary and metastatic tumours (Caverzasi et al., 2016;Kadota et al., 2020;Mao et al., 2020), as well as regions of oedema withintumours (Masjoodi et al., 2018;Okita et al., 2023), andtumour grading (Figini et al.,2018;Maximov et al.,2017;Wen et al.,2015;Zhao et al.,2018).

NODDI is still an emerging technology, akin to contemporary compartmental models ofDWI. Currently, the existing NODDI model (Zhang et al., 2012) does not account for differences inT2 relaxation between the model compartments. Therefore, when differences incompartmental T2 exist, it is difficult to disentangle differences in diffusion fromrelaxation. This results in suboptimal estimation of the NODDI parameters, withoverestimation of the free-water volume fraction leading to inaccuracies inmeasurements of NDI and ODI (Bouyagoubet al., 2016), potentially impacting the application of NODDI toconditions with T2 changes, such as in tumour tissue (Lampinen et al., 2019;Wen et al., 2015).Gong et al. (2020)recently proposed the multiple echotime NODDI (MTE-NODDI) technique, which provides robust estimates of thenon-T2-weighted NODDI parameters and compartment-specific T2 values from diffusiondata acquired at multiple echo times. With its ability to provide the same NODDImeasures with improved parameter precision, MTE-NODDI may enhance the conspicuity ofneuropathology, including imaging of brain tumours and the invading margin.

Therefore, in this study, we investigate the effectiveness of MTE-NODDI as anon-invasive*in vivo*imaging technique to improve detection of thetumour margin and demonstrate sensitivity of the technique to infiltrating tumourcells in a well-characterised patient-derived xenograft mouse model of GBM. Wefurther report correlations of MTE-NODDI parameters with fluorescence intensity fromgreen fluorescent protein (GFP) positive tumour cells and evaluate thetechnique’s specificity to tumour-induced microstructural pathology.

## 2 Materials and Methods

All procedures were performed in compliance with the Animal Scientific ProceduresAct, 1986 and approved by the UCL Animal Welfare and Ethical Review Body (AWERB) inaccordance with the international guidelines of the Home Office (UK).

### 2.1 Glioblastoma mouse model

A patient-derived xenograft mouse model of GBM was used in the current study. Thepatient (G144) was diagnosed with malignant astrocytoma (GBM), with increasedcopy numbers of chromosome 7 (EGFR genes map to this chromosome) (Pollard et al., 2009).Xenografts were performed on CD-1 nude mice using the G144 cell line which showspropensity for invasion through the white matter into the contralateralhemisphere. The tumour model utilised in this study was originally establishedin female mice (Brooks et al.,2021;Pollard et al.,2009) and, as such, only female mice were used. 8–12 week-oldimmunocompromised mice (n = 7) (purchased from Charles RiverLaboratories) underwent stereotactic implantation of 1 x 10^5^GFP-labelled G144 cells (anteroposterior 0, mediolateral -2.5, dorsoventral -3).The tumour model used in this study shows high latency with all the micepresenting tumours and they were imaged at three timepoints post injection; 4,6, and 8 weeks, following which they were sacrificed. A separate group of age-and strain-matched naïve mice (n=3) were used in a controlexperiment and underwent the same imaging protocol as above.

### 2.2 Animal preparation for MRI

Prior to commencing the MRI acquisitions, the mice were placed in an inductionchamber and anesthetised with inhaled isoflurane (2% isoflurane at 1 l/minO_2_) until withdrawal reflex was lost. They were then transferredinto the MRI mouse cradle with bite bar, nose cone, and ear bars to ensure awell-secured head position to minimise motion artifacts. Eye ointment wasapplied to prevent drying. Temperature and breathing rate were monitoredthroughout all the experiments using a rectal probe and a respiration pad (SAInstruments). Core body temperature was maintained at 37 ± 0.5°Cvia regulation of an adjustable temperature water bath. Isoflurane level wasmanually adjusted throughout the duration of the scan to maintain a consistentrespiration rate of ~100 bpm.

### 2.3 Mouse MRI protocols

Images were acquired on a 9.4 T Bruker imaging system (BioSpec 94/20 USR) with ahorizontal bore and 440 mT/m gradient set with an outer/ inner diameter of 205mm/116 mm, respectively (BioSpec B-GA 12S2), 86 mm volume coil, and afour-channel array receiver-surface coil (RAPID Biomedical GmbH) for thetransmission and reception of RF signal. Tumours were localised using astructural T2-TurboRARE sequence (fast-spin echo, Paravision v6.0.1). Theolfactory bulbs were used as an anatomical landmark to maintain consistency inslice positioning between subjects, and the slices covered the cortex and allsubcortical structures up to the cerebellum. Imaging parameters for theT2-weighted acquisition were as follows: TR = 4000 ms, TE = 45 ms,FOV = 21 x 16 mm^2^, data matrix 256 x 196, and 14 x 600µm coronal slices.

For MTE-NODDI, DWI images were acquired using a 4-shot spin echo-planar imaging(EPI) sequence. Imaging parameters were: 14 slices, slice thickness = 600µm; FOV = 20 x 16 mm^2^; data matrix 110 x 85; andTR= 2500 ms. To implement MTE-NODDI, the DWI images were acquired atthree different echo times of 30, 45, and 60 ms. At each echo time, MRI protocolconsisted of two shells, detailed as follows:

1. Shell One: 30 directions, five b = 0 s/mm^2^images, anddiffusion weighting of b = 2000 s/mm^2^
2. Shell Two: 15 directions, five b = 0 s/mm^2^images, anddiffusion weighting of b = 700 s/mm^2^

with gradient duration and separation δ∕Δ =4.5∕11 ms for all b-values and TE’s. The overall acquisition timefor each mouse per imaging session was ~120 minutes.

### 2.4 Image processing and modelling of diffusion data

The effects of noise and imaging artifacts on the DWI images were reduced byapplying a denoising method based on the random matrix theory (MRtrix3) (Veraart et al., 2016),correction of B0 inhomogeneity, and motion with TOPUP (Andersson & Sotiropoulos,2016) in FMRIB Software Library (FSL, University of Oxford, UK). TheT2-weighted and DWI acquisitions were then co-registered to a referenceb=0 image. T2-weighted images were resampled to match the DWI images inAFNI (Analysis of Functional NeuroImages, National Institutes of Health, USA),and brain masks were created manually on T2-weighted images using ITK-SNAP. Meandiffusivity (MD) measures were generated from diffusion data obtained at TE= 30 ms using dtifit in FSL, which fits a diffusion tensor model at eachvoxel of the data that has been pre-processed. NODDI parameters were estimatedfor each TE session separately with the NODDI MATLAB Toolbox, and the estimatedparameters were aligned to the first TE session to extract TE-independentMTE-NODDI parameters (Gong et al.,2020).

### 2.5 Selection of tumour regions of interest

For quantitative analysis, tumour regions of interest (ROIs) were manuallydefined on T2-weighted images (T2WI) using ITK-SNAP by the two authors (SK andDG) who were blinded to the other’s results. The images and ROIs werepresented to a consulting neurosurgeon (CSH) for approval. In our experiencewith the G144 model, tumour cells exhibit a spread beyond the tumour bulk,infiltrating into the peritumoural regions of corpus callosum, striatum, andcortex regions (Brooks et al.,2021). Therefore, the following ROIs were selected:

#### 2.5.1 Tumour bulk

Regions with hyperintense signal in the ipsilateral hemisphere on the coronalT2WI were identified as tumour bulk.

#### 2.5.2 Peritumoural margin

Regions outside the tumour bulk, where there are no T2WI changes, weredefined in the ipsilateral hemisphere in three distinct anatomicalregions—corpus callosum, striatum, and cortex. Given that the G144model replicates the GBM feature of invasion along the corpus callosum, amargin region in the corpus callosum adjacent to the tumour bulk was alwaysselected. ROIs were adjusted to exclude any confounding anatomicalstructures (e.g., the lateral ventricle in the striatal ROI, grey matterstructures in the corpus callosum ROI, etc.). These exclusion criteriaresulted in ROIs of the following sizes: corpus callosum—1 pixel(~0.182 mm) in the xy direction; striatum and cortex—up to 3 pixels(~0.550 mm). As can be seen in the representative images presented in thismanuscript (Fig. 2),confounding anatomical structures such as external capsule may contaminatestriatal ROIs (or corpus callosum in the cortical ROI) if extended beyond 3pixels. In the contralateral hemisphere, ROIs equivalent to the peritumouralmargins were defined as controls for comparison. Subsequently, the same ROIswere copied onto diffusion MRI images and mean values for NDI,*f_en_*, ODI, and MD were extracted forquantitative analysis.

#### 2.5.3 Histology ROI for correlation

**Fig. 1.**
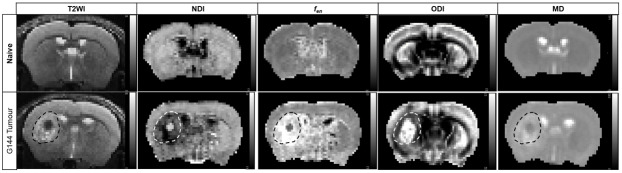
Representative coronal T2WI images and maps of MTE-NODDI NDI,*f_en_,*ODI, and DTI MD. Top panel:representative maps from a naive mouse at the third timepoint showing nodiscernable changes across both hemispheres. Bottom panel: maps from aG144 tumour-bearing mouse at the third timepoint revealing changes dueto tumour cells infiltrating into the peritumoural margins. Tumour bulkidentified on the T2WI is indicated by a dashed line on all maps.

Due to fixation and shrinkage of tissue during histology, a pixel-by-pixelcomparison was not possible across the MRI and histology data sets. As such,ROIs encompassing the cortex across both hemispheres of the brain werechosen to enable an unbiased comparison between the two sets of images(Supp.Fig. 1). In animals where tumour bulk was identified in thecortex, these pixels were excluded from both the MRI and GFP images toensure accurate correlation analysis. Opting for an ROI covering the entirecortex simplifies delineation, while simultaneously mitigating any artifactsand inaccuracies caused due to tissue fixation.

### 2.6 Quantification of GFP+ fluorescence from tumour cells

G144 tumour cells were transduced to constitutively express GFP using alentiviral approach. Lentivirus carrying the GFP transgene was produced bycotransfecting with the HIV-1 packaging vector Delta8.9 and the VSVG envelopeglycoprotein into 293 T cells using polyethylenimine. Virus was concentrated byultracentrifugation (3 h, 50,000 × g, 4°C) (Brooks & Parrinello,2017). For quantification of the fluorescence intensity fromGFP+ tumour cells, mice were perfused with 4% PFA and the brainpost-fixed in 4% paraformaldehyde overnight at 4°C. Two explanted brainsfrom the MRI-imaged mice were assigned for downstream mechanistic analyses and,as such, were not included in this study, rendering them unavailable forimmunofluorescence. From the remaining mice (n = 5), 50 μmvibratome sections were cover-slipped, dried, and imaged using a Leica DMi8Inverted microscope (Leica Microsystems, Wetzlar, Germany). Immunofluorescenceimages from each mouse (n = 5; 1 slice per mouse) were individuallyimported into ImageJ, and ROIs encompassing the entire cortex were drawn. Imageswere then thresholded by intensity to eliminate any signal from background, andsignal intensity from GFP-positive tumour cells within the ROI was quantified as% Area under fluorescence and exported into GraphPad Prism for correlation withMRI measurements.

### 2.7 Statistical analysis

Statistical analysis of the data was performed using GraphPad Prism 9.0. All dataare expressed as mean ± SD and were tested for normality usingShapiro-Wilk test. For the intra-animal changes between contralateral andperitumoural margin regions, paired t-tests were used to test for statisticallysignificant difference. For the longitudinal study, we reported the data as mean± SD of paired differences in all animals at each timepoint. In addition,Pearson correlation analysis was used to determine the correlation between MRIparameters and GFP+ fluorescence intensity from tumour cells. For allstatistical tests, the significance level was p < 0.05.

## 3 Results

### 3.1 Application of MTE-NODDI to mouse model of glioblastoma

We probed the invading margin of GBM using MTE-NODDI (Gong et al., 2020), a recentimprovement of the conventional NODDI model (Zhang et al., 2012), which enables us to estimatenon-T2-weighted NODDI parameters such as NDI,*f_en_*,and ODI. A well-characterised G144 patient-derived xenograft mouse model, whichrecapitulates the hallmark feature of GBM to invade to the contralateralhemisphere along the white matter tracts of the corpus callosum (Brooks et al., 2021;Pollard et al., 2009), wasused in combination with MTE-NODDI.

To assess the sensitivity of MTE-NODDI to the spread of tumour cells,GFP-labelled G144 cells were stereotactically injected into the striatum ofimmunocompromised mice and imaged longitudinally on the 9.4T MRI system at threetimepoints after injection (4, 6, and 8 weeks). Previous studies suggest thatthe invasive cell fates and invasion patterns of G144 cells were different indifferent microenvironments of the brain (Brooks et al., 2021). In line with theseobservations, at the three timepoints, we used T2WI images to definehyperintense tumour bulk and peritumoural margin regions extending into thecorpus callosum as a region of white matter, as well as cortex and striatum asregions of grey matter for quantitative analysis using MTE-NODDI.

**Fig. 2.**
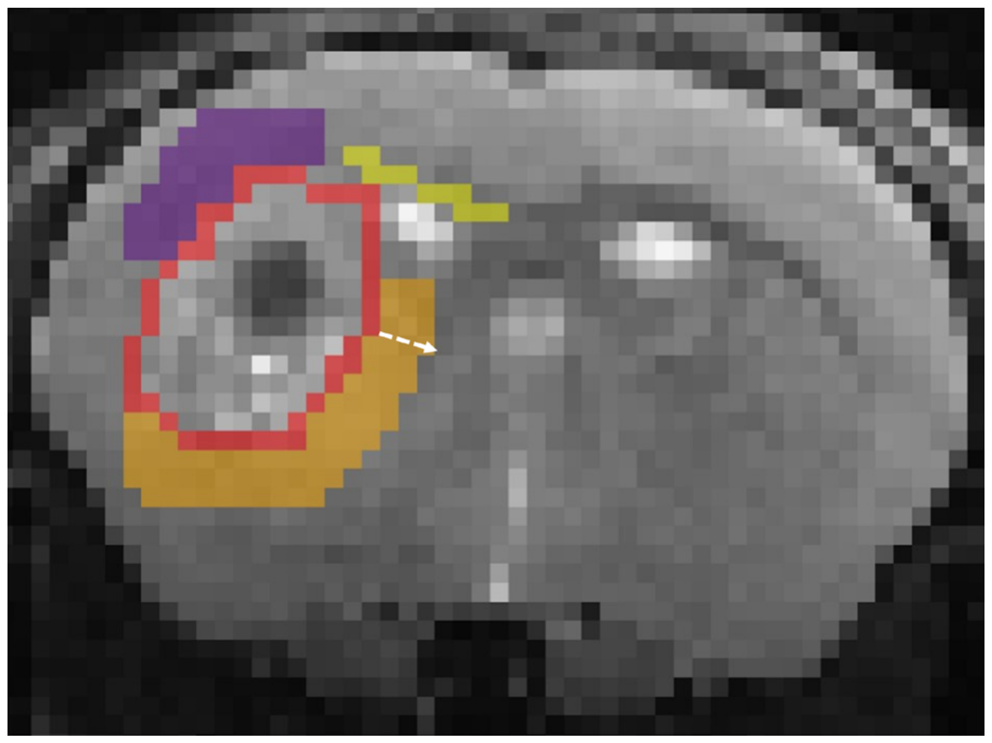
Representative coronal T2WI image with defined tumour regions of interest(ROIs) for quantification of MRI parameters. For illustrative purposes,outline of tumour bulk defined on T2WI is shown in red; the peritumouralmargin regions in the cortex, corpus callosum, and striatum aredisplayed in purple, yellow, and orange respectively. White arrowillustrates the 3-pixel extension of the ROIs around the tumourbulk.

Additionally, to ascertain whether MTE-NODDI provides improved sensitivity toinfiltrating GBM cells, we also applied a standard diffusion tensor imaging(DTI) model to the diffusion data to calculate maps of MD.Figure 1presents images of naïve andG144 tumour-bearing mice from a bespokeT2WI sequence, together with concomitantmaps of NDI,*f_en_*, ODI, and MD. The tumour bulk isreadily identified as a hyperintense area on the T2WI image. The peritumouralmargin regions (areas without T2 changes) surrounding the hyperintense bulk(Fig. 2) are located inthe cortex, corpus callosum, and striatum.

### 3.2 Peritumoural margin detection using MTE-NODDI

**Fig. 3.**
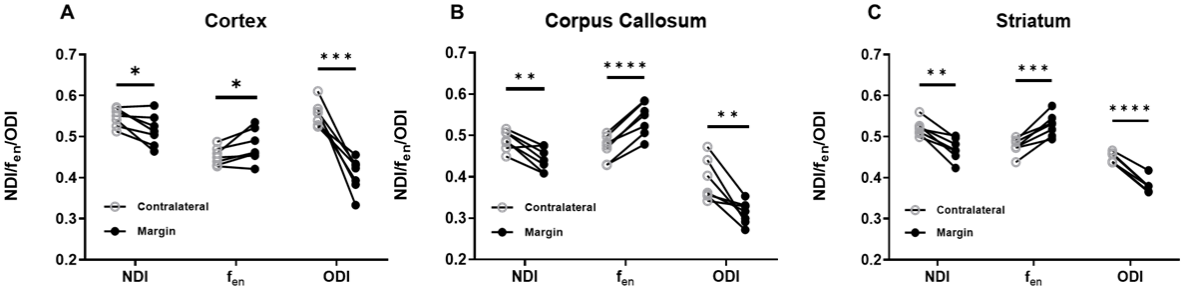
Mean values of MTE-NODDI parameters extracted from the contralateral andperitumoural margin ROIs for each animal (n=7) at the 8-weektimepoint. NDI,*f_en_*, and ODI values from thecontralateral and peritumoural margins in (A) Cortex, (B) Corpuscallosum, and (C) Striatum. Each data point on the plots represents themean value for individual mouse. Paired t-tests were used to teststatistically significant differences between contralateral andperitumoural margins (*p < 0.05, **p< 0.005, ***p < 0.001,****p < 0.0005).

A focus of this study was to investigate the conspicuity of the MTE-NODDIparameters to peritumoural margin regions beyond the tumour bulk, where nodiscernable changes were present in the T2WI images. At the 8-week timepoint,when the tumour is most developed, comparison of the MTE-NODDI parametersbetween the peritumoural margin and equivalent contralateral regions (cortex,corpus callosum, and striatum) revealed marked changes in NDI,*f_en_*_,_and ODI values outside thetumour bulk (Fig. 3,Supp. Fig.2).

In the cortex, all three MTE-NODDI parameters NDI (p < 0.05),*f_en_*(p < 0.05) and ODI (p <0.0005) demonstrated clear differences in the peritumoural margin; most notablyin the ODI (Fig. 3A,Supp. Fig.2A). Similarly, in the corpus callosum margin, NDI (p <0.005), ODI (p < 0.01), and*f_en_*(p <0.0001) values demonstrated excellent sensitivity of MTE-NODDI to detect tumourmargin in both grey and white matter regions outside the bulk (Fig. 3B,Supp. Fig.2B). As in the corpus callosum and cortex, peritumoural margin ROIsin the striatum had a lower ODI (p < 0.0001), NDI (p < 0.005) andhigher*f_en_*(p < 0.001) values (Fig. 3C,Supp. Fig.2C).

Gadolinium-enhanced T1 images, alongside T2 images, are used clinically to definethe area of tumour bulk (Price& Gillard, 2011). Due to the limited time during the MRIacquisition protocol, we did not acquire post-contrast T1 images. Therefore, ina separate group of tumour-bearing animals (n = 2), we compared themeasured tumour volumes in our model of GBM using both gadolinium-enhancedT1-weighted and the T2WI used to identify tumour bulk. The tumour volumes werecomparable (mean percentage difference = 6.7%), supporting the use ofT2WI to define tumour bulk in our model (Supp. Fig. 3).

**Fig. 4.**
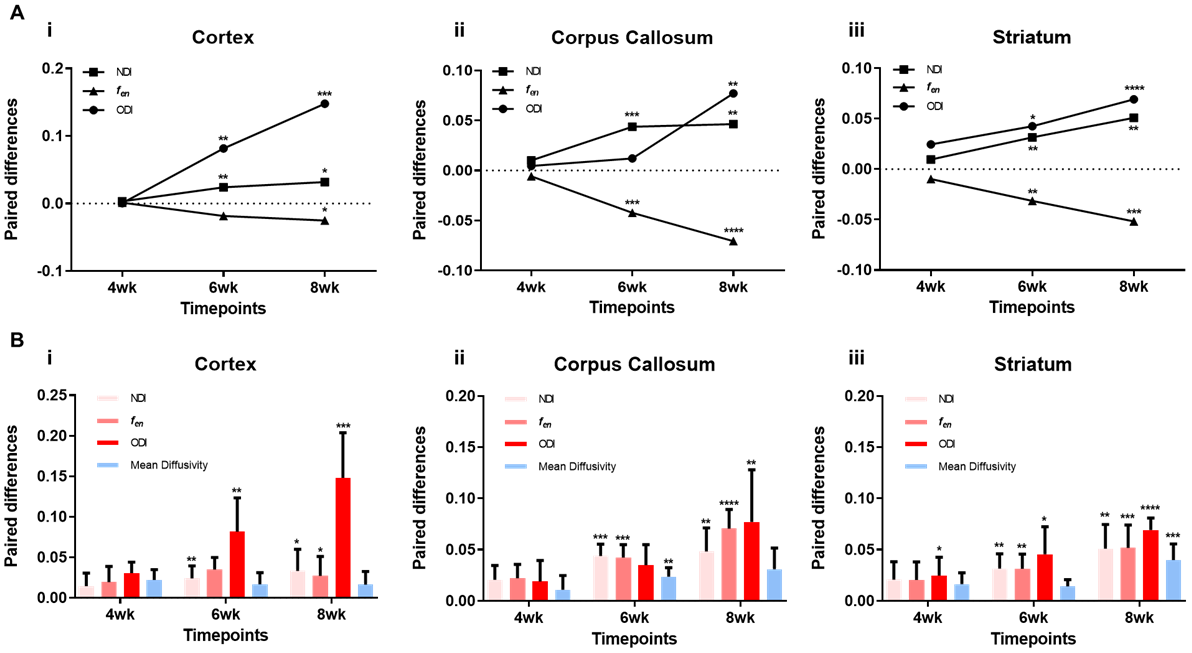
MTE-NODDI and MD changes over time with developing tumour. (A) Paireddifferences (Contralateral–Peritumoural Margin) of NDI,*f_en_*, and ODI at 4 wk, 6 wk, and 8 wktimepoints in (i) Cortex, (ii) Corpus callosum, and (iii) Striatum. Eachdata point in the plot represents the mean of paired differences fromall animals (n=7). (B) Paired differences(Contralateral–Peritumoural Margin) of NDI,*f_en_*, ODI, and MD at 4 wk, 6 wk, and8 wk timepoints in (i) Cortex, (ii) Corpus callosum, and (iii) Striatum.Barplots represent means of paired differences from all animals(n=7), where MTE-NODDI parameters are shown in red, and DTI MD isplotted in blue for illustrative purposes. Paired t-tests were used totest statistically significant differences between contralateral andperitumoural margins (*p < 0.05, **p< 0.005, ***p < 0.001,****p < 0.0005).

When defining the equivalent control ROIs (bulk, peritumoural margins in thecortex, striatum, and corpus callosum) in the contralateral hemisphere, therecould be slight variations in ROI placement due to the tumour mass in theipsilateral hemisphere. As such, additional controls were performed in a groupof age- and strain-matched naïve mice to assess the effect of the smallvariation in ipsi- and contralateral ROIs (n=3) placement, due to thetumour mass in the ipsilateral striatum, on the MRI data. The MRI images fromthe naïve mice were co-registered to the images from the tumour-bearingmice using FMRIB Software Library (FSL, University of Oxford, UK). Subsequently,the ROIs drawn on tumour-bearing mice were superimposed onto images from thenaïve mice and mean values for NDI,*f_en_*, ODI,and MD were extracted for quantitative analysis. We did not note any differencesin NDI,*f_en_*, and ODI values between ROIs due to thetumour (Supp.Fig. 4). Therefore, there is limited contribution to the peritumouralmargin data from the regional background variation in the selected ROIs.

### 3.3 Monitoring tumour invasion into peritumoural regions

We next assessed the sensitivity of MTE-NODDI parameters to tumour-inducedmicrostructure changes (NDI,*f_en_*, and ODI) at allthree imaging timepoints (4, 6, and 8 weeks) across three anatomical regions(Fig. 4A). MTE-NODDIparameters measured from ROIs drawn at the respective timepoints were clearlysensitive to tumour evolution over the 4-week imaging period. These peritumouralmargin changes were most notable from the 6-week time point onwards. Forinstance, beginning at the 6-week timepoint, all MTE-NODDI parameters in thestriatum exhibited sensitivity to tumour-induced pathology, with decreased NDI(p < 0.005), and ODI (p < 0.05), while the*f_en_*(p < 0.005) was increased (Fig. 4Ai-iii).

### 3.4 Increased sensitivity of MTE-NODDI to invading tumour margin compared with MD

Our subsequent aim was to evaluate whether MTE-NODDI was more sensitive totumour-induced changes than the established DWI measure of MD. We observed thatdifferences in NDI,*f_en_*_,_and ODI valueswere consistently higher than MD values, indicating that MTE-NODDI offersincreased sensitivity to microstructural changes in the peritumoural margin asthe tumour invades normal brain tissue. More specifically, we detected increasesin striatal ODI as early as week 4 without any associated changes in MD (Fig. 4B). This heightenedsensitivity was also observed at 6 and 8 weeks in the cortex, as well as at 8weeks in the corpus callosum, thereby providing strong evidence for theincreased conspicuity of MTE-NODDI to tumour-induced microstructural changes inregions without MD or T2WI changes. Importantly we did not observe any changes(NDI,*f_en_*_,_ODI, and MD) in controlage-matched and strain-matched naïve animals (n=3) over the 3timepoints (Supp. Fig. 5). Taken together, this provides evidence that MTE-NODDIparameters are sensitive to tumour-induced microstructural pathology as itinvades into regions outside the tumour bulk.

### 3.5 Invading tumour cells correlate with NDI and *f _en_*

**Fig. 5.**
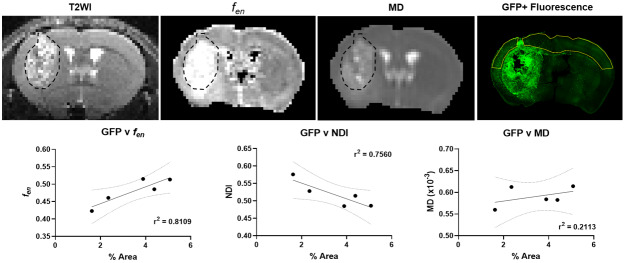
MTE-NODDI values in the cortex correlated with GFP+ fluorescencefrom tumour mice. Top Panel: Representative T2WI image,*f_en_*map, MD map, and GFP+histological image from a G144 tumour mouse. Tumour bulk as identifiedon the T2WI is indicated by a dashed black line. The cortical ROI usedfor correlation is indicated in solid yellow. Bottom Panel: Correlationof*f_en_*, NDI, and MD to GFP+fluorescence. MTE-NODDI and MD values were extracted from a region drawnin the cortex across both hemispheres. GFP+ fluorescence iscalculated as % Area under fluorescence from an equivalent corticalregion drawn on immunohistological images (n=5). Pearson’scorrelation coefficient was used to investigate a possible correlation(r^2^values reported on respective plots).

Finally, we sought to examine the effectiveness of MTE-NODDI as an*invivo*imaging marker to detect invading tumour cells in the tumourmargin, by comparing fluorescence (% area) from GFP+ tumour cellsmeasured using immunohistological images with the MRI parameters (Fig. 5). Importantly, theGFP+ cell distribution observed in the immunohistological imagesconfirmed tumour cell infiltration into all three anatomical brain regionstested (cortex, corpus collosum, and striatum) (Fig. 5; top panel). MTE-NODDI values in thecortex show that both*f_en_*(R^2^=0.81 and p < 0.05) and NDI (R^2^= 0.76 and p <0.05) demonstrated strong correlations with GFP+ fluorescence, with MDdisplaying no such correlation (R^2^= 0.21 and p =0.44). As such, we observe improved specificity of the technique over MD fordetecting tumour-induced pathology correlating with migrating tumour cells. Toexpand upon these findings, we analysed an additional 9 GFP+immunohistological sections from the rostral edge of the tumour bulk. We did notfind any correlations between GFP+ fluorescence and the MRI parameters.However, in this anterior region, the cell count was eight times lower (0.28%)compared to the central, more cellularly dense portion of the tumour (1.56%) (p= 0.05) (Supp. Fig. 6). We believe the lack of correlation is due to thescarcity of cells thus limiting sensitivity, as most likely beyond the limits ofdetection in the regions at the anterior boundary of the tumour.

## 4 Discussion

Precise tumour margin delineation in GBM remains a profound challenge due to itshighly invasive nature and diffuse pattern of tumour cell migration into healthybrain regions. Here, we report the first application of MTE-NODDI to non-invasivelydetect infiltrating cells in a patient-derived xenograft mouse model of GBM inregions outside the tumour bulk. We demonstrate that MTE-NODDI detectedtumour-induced pathology in margin regions without T2 or MRI-diffusion changes.Furthermore, MTE-NODDI parameters correlated with immunohistological measure oftumour cell infiltration. These findings highlight the sensitivity of MTE-NODDI toinvading tumour cells and associated pathology, suggesting its utility in imagingperitumoural margins of GBM.

Traditionally, NODDI was developed to provide microstructural information that ismore specific than DTI by separating the diffusion signal into intra-neurite,extra-neurite, and free water fractions (Zhang et al., 2012). The NODDI model, generates two keyvariables, neurite density index (NDI) and orientation dispersion index (ODI).Additionally, free water fraction*(f_iso_)*andextra-neurite volume fraction (*f_en_*) can be derived whenstudying the influence of CSF and extra-neurite structures (like soma, glial cells,invading tumour cells etc.) respectively. The technique is suitable for both greyand white matter (Winston et al.,2014) and has been valuable in evaluating axonal/dendritic degenerationand fiber orientation in various neurological diseases, such as Alzheimer’sdisease (Colgan et al., 2016;Fu et al., 2020;Parker et al., 2018),Parkinson’s disease (Kamagata etal., 2017;Mitchell et al.,2019), MS (Schneider et al.,2017;Spano et al.,2018), ischemic stroke (Mastropietro et al., 2019;Z. Wang et al., 2019), and epilepsy (Sone et al., 2018;Winston et al., 2014). Importantly, NODDI has also beensuccessfully applied to brain tumours (Kamiya et al., 2020;Sanvito et al., 2021), primarily in grading (Figini et al., 2018;Zhao et al., 2018) but also todifferentiate primary GBM tumour bulk from brain metastases (Kadota et al., 2020;Mao et al., 2020;Wen et al., 2015), as well asvasogenic oedema from tumour infiltration (Caverzasi et al., 2016;Chong et al., 2021;Masjoodi et al., 2018). However, there are no reportsinvestigating the role of NODDI in detecting infiltrating tumour margin beyondregions with T1 or T2 abnormalities.

Outside the bulk of the tumour, in the hard-to-detect peritumoural margin regions, weobserve marked changes in the MTE-NODDI parameters. Several studies have examinedthe association between mean diffusivity (MD), also known as the apparent diffusioncoefficient (ADC), and tumour cellularity in GBM. Some studies have reported adecrease in MD with tumour progression and a negative correlation between MD andtumour cellularity in these regions (Chang et al., 2017;Chenevert et al., 2000;Kono et al., 2001;Sugaharaet al., 1999), while others have found MD maps less useful or reportedinverse findings (Berro et al.,2019;Price et al.,2006;Sadeghi et al.,2008). When we compared our measurements of MTE-NODDI changes to the moreestablished MD, we observed that MTE-NODDI has a greater sensitivity to infiltratingtumour cells. In our mouse model, we observed an increase in MD values in theperitumoural margins, suggesting the influence of other factors such as peritumouraloedema (Lemercier et al., 2014).Given that the hyperintense regions in T2WI and contrast-enhancing regions inT1-weighted imaging were comparable, this finding suggests that the model does notexhibit oedema. The absence of oedema is reinforced by the lack of hyperintensesignals on T2WI outside the boundaries of the contrast-enhancing region. Ourfindings suggest that infiltrating tumour cells, perhaps coupled withmicrostructural changes induced by cell invasion, can be detected by MTE-NODDI. Assuch, MTE-NODDI with its improved sensitivity may provide a tool for detectingtumour invasion into the peritumoural margin regions in GBM.

In terms of tumour growth, MTE-NODDI parameters readily detected tumour evolution(4–8 weeks) as the tumour cells infiltrate out from the bulk into theperitumoural margins. NODDI has been previously used to investigate the relationshipbetween disease progression and WM-changes in traumatic brain injury (Palacios et al., 2020) stroke(Mastropietro et al., 2019),athletic concussion (Churchill et al.,2019), and schizophrenia (Kraguljac et al., 2021). Taken together with our data, we anticipatethat MTE-NODDI could be valuable in evaluating longitudinal microstructural changesin the brain induced by infiltrating tumour cells.

Previous studies have noted increased ODI in the tumour bulk, likely due todisruption of neuronal structural integrity (Masjoodi et al., 2018;Onishi et al., 2022). Conversely, we observe a decreasein ODI in the margin regions, which may suggest increased microstructuraldirectionality due to invading GBM tumour cells. Recently, invasion in GBM has beendescribed as having multiple traits of neuronal development, including formation ofneurogliomal synapses that simulate ‘neurite-like’ tumour microtubes(Venkataramani et al.,2019), alongside increases in solid stress and compression (Seano et al., 2019), which maylead to a decrease in dispersion of neurites and consequently lower ODI. Thus, it isconceivable, that in the peritumoural margin regions, tumour cell infiltration maycontribute to the observed decrease in ODI values.

Our observed increase in*f_en_*values may be driven byinfiltrating tumour cells within the extra-neurite spaces in the peritumouralmargin. When patient-derived glioma cells are implanted into rodent brains, asubstantial amount of these cells migrate along the vascular surfaces to invade thehealthy brain (Brooks &Parrinello, 2017;Cuddapahet al., 2014). These infiltrating tumour cells can be associated with theNODDI extra-neurite compartment (*f_en_*), in the samefashion as microglia, glial cells, and ependymal cells (Caverzasi et al., 2016;Kadota et al., 2020). Previously, it has beendemonstrated that microglial density is a key contributor to measures of NODDIextra-neurite compartment (*f_en_*) (Yi et al., 2019). Similarly, asGBM cells infiltrate into the peritumoural margins with tumour development, there isa corresponding increase in*f_en_*values supporting thecontribution of GBM cells to an increase in the measured extra-neurite compartmentindex (*f_en_*).

We also observed lower NDI in the peritumoural regions, which may be due to arelative decrease in intra-neurite volume fraction caused by infiltrating tumourcells in the extra-neurite spaces, causing a shift in diffusion property of thetissue from a restricted intra-neurite compartment towards a hindered extra-neuritecompartment (Kadota et al.,2020;Yi et al., 2019).Therefore, as tumour cells invade into the margin, this may reduce the intra-neuritevolume fraction leading to a decrease NDI. Previous studies featuring use of NODDIin the glioma bulk (Jin et al.,2020;Kadota et al.,2020;Onishi et al.,2022;Zhao et al.,2018) have shown a decreased NDI, suggesting possible neuronal loss andfibre disruption, yet this may also reflect the increased cellular density in thetumour bulk, as observed in the margin of our study.

These observations were further explored by investigating the relationship betweenMTE-NODDI parameters and infiltrating tumour cells (GFP+ fluorescence). Inour tumour model,*f_en_*and NDI correlated well withGFP+ fluorescence. NODDI has been previously used to investigatemicrostructural alterations in an Alzheimer’s mouse model of tau pathology,where NDI values correlated with disease burden (Colgan et al., 2016). Our results indicate thatinfiltrating tumour cells may account for the changes in MTE-NODDI parameters,although this does not exclude additional tumour-induced microstructural changes,such as pathological demyelination (Brooks et al., 2021;J.Wang et al., 2018), and water retention in the surrounding tissue (Onishi et al., 2022;Wen et al., 2015).

As this was a proof-of-concept study, there is an opportunity for building on theexperimental design. The potential presence of tumour cells within the contralateralcontrol hemisphere may result in an underestimation of the observed differencesbetween the peritumoural and contralateral control ROIs. Incorporating a baselinescan to establish control values before the onset of disease may contribute to theability of MTE-NODDI to detect peritumoural invasion. There is also an opportunityto further disentangle the observed differences in MTE-NODDI metrics resulting frominfiltrating tumour cells and those caused due to pathological microstructuralchanges such as demyelination, angiogenesis, alterations to the extra cellularmatrix, and other deformations in the brain due to tumour-growth (Brooks et al., 2022;Lathia et al., 2011;Seano et al., 2019). Furthermore,a multi-timepoint acquisition of MRI data with matched multi-slice histology at eachtimepoint would enable a comprehensive understanding of the specificity of MTE-NODDImetrics to infiltrating tumour cells across the entire brain. Investigating thehistological images from the anterior portion of the tumour did not yieldsignificant findings, which we believe is likely due to the limited number ofinvading tumour cells in that region (8 times lower compared to the central core ofthe tumour). In addition, the relatively small size of the tumour compared to theMRI slice thickness may result in small or subtle areas of tumour infiltration to bemissed or averaged out in the imaging data, reducing the ability to preciselycorrelate the MRI findings with the histological data. Future studies shouldconsider using reduced imaging slice thickness and multiple histological sectionsspanning the entire tumour length to further establish specificity of the techniquefor detecting migrating tumour cells. Additionally, establishing directco-registration between MRI images and histological sections could facilitate apixel-by-pixel analysis of the MRI signal, which was not possible in this study.Moreover, in the present study, we define the tumour bulk and peritumoural marginregions based on T2-weighted images. To better align with imaging protocols commonlyemployed in clinical settings, a multi-parametric acquisition that includescontrast-enhanced T1-weighted images and FLAIR imaging for ROI delineation, alongwith T2 mapping for quantitative analysis, would be more suitable. In terms of othermethods, the ongoing development of imaging techniques (Laack et al., 2021;Laprie et al., 2024;Ramesh et al., 2022) suggests that a multiparametricimaging approach may contribute to understanding this complex and challenging braintumour type.

## 5 Conclusions

In conclusion, this study establishes the clear value of MTE-NODDI in detectinginfiltrating GBM cells in the peritumoural margin outside regions of T2WI and MDabnormalities. MTE-NODDI parameters NDI,*f_en_*, and ODIall change markedly in areas with tumour cell infiltration and associatedmicrostructural alterations. Moreover, the correlations between immunohistologicalmeasure of tumour cells and MTE-NODDI parameters provide evidence of sensitivity ofMTE-NODDI to low numbers of infiltrating tumour cells. Mounting evidence showsimproved survival with extending resection into the non-contrast enhancing regions(De Leeuw & Vogelbaum,2019;Jackson et al.,2020). Therefore, findings from this study suggest that MTE-NODDI couldbe used to improve tumour margin detection and aid neurosurgeons in maximising theextent of surgical resection to achieve better survival and quality-of-lifeoutcomes.

## Data and Code Availability

The data generated in this study are available upon reasonable request from thecorresponding author.

## Author Contributions

S.R.K.: Data curation, Formal analysis, Investigation, Methodology, Visualisation,and Writing—original draft. D.G.: Data curation, Formal analysis,Investigation, Visualisation, and Writing—review & editing. D.T.: Datacuration, Methodology, Software, and Writing—review & editing. L.J.B.:Methodology, Writing—review & editing. T.G.: Methodology, Validation,and Software. A.C.S.J.: Formal analysis, Visualisation, and Writing—review& editing. C.S.H.: Formal analysis, Writing—review & editing.M.C.: Formal analysis, Writing—review & editing. T.L.K.: Formalanalysis, Writing—review & editing. J.A.W.: Methodology,Writing—review & editing. D.J.S.: Formal analysis,Writing—review & editing. L. T.: Formal analysis,Writing—review & editing. V.S.: Formal analysis, Supervision, Fundingacquisition, and Writing—review & editing. S.M.: Formal analysis,Supervision, Funding acquisition, and Writing—review & editing. H.Z.:Metholodology, Validation, and Software. S.P.: Conceptualisation, Formal analysis,Supervision, Funding acquisition, and Writing—review & editing.M.F.L.: Conceptualisation, Formal analysis, Supervision, Funding acquisition, andWriting—original draft.

## Declaration of Competing Interest

The authors declare that they have no competing interests.

## Supplementary Material

Supplementary Material

## References

[b1] Andersson , J. L. R. , & Sotiropoulos , S. N. ( 2016 ). An integrated approach to correction for off-resonance effects and subject movement in diffusion MR imaging . NeuroImage , 125 , 1063 – 1078 . 10.1016/J.NEUROIMAGE.2015.10.019 26481672 PMC4692656

[b2] Basser , P. J. , Mattiello , J. , & LeBihan , D. ( 1994 ). MR diffusion tensor spectroscopy and imaging . Biophysical Journal , 66 ( 1 ), 259 – 267 . 10.1016/S0006-3495(94)80775-1 8130344 PMC1275686

[b3] Berro , D. H. , Collet , S. , Constans , J. M. , Barré , L. , Derlon , J. M. , Emery , E. , Guillamo , J. S. , & Valable , S. ( 2019 ). Comparison between MRI-derived ADC maps and 18FLT-PET in pre-operative glioblastoma . Journal of Neuroradiology , 46 ( 6 ), 359 – 366 . 10.1016/J.NEURAD.2019.05.011 31229576

[b4] Bouyagoub , S. , Dowell , N. , Hurley , S. A. , Wood , T. C. , & Cercignani , M. ( 2016 ). Overestimation of CSF fraction in NODDI: Possible correction techniques and the effect on neurite density and orientation dispersion measures. * 24 ^th^ Annual Meeting & Exhibition * . Archive.Ismrm.Org . https://archive.ismrm.org/2016/0007.html

[b5] Brooks , L. J. , Clements , M. P. , Burden , J. J. , Kocher , D. , Richards , L. , Devesa , S. C. , Zakka , L. , Woodberry , M. , Ellis , M. , Jaunmuktane , Z. , Brandner , S. , Morrison , G. , Pollard , S. M. , Dirks , P. B. , Marguerat , S. , & Parrinello , S. ( 2021 ). The white matter is a pro-differentiative niche for glioblastoma . Nature Communications , 12 ( 1 ), 1 – 14 . 10.1038/s41467-021-22225-w PMC8042097 33846316

[b6] Brooks , L. J. , & Parrinello , S. ( 2017 ). Vascular regulation of glioma stem-like cells: A balancing act . Current Opinion in Neurobiology , 47 , 8 – 15 . 10.1016/J.CONB.2017.06.008 28732340

[b7] Brooks , L. J. , Simpson Ragdale , H., Hill , C. S. , Clements , M. , & Parrinello , S. ( 2022 ). Injury programs shape glioblastoma . Trends in Neurosciences , 45 ( 11 ), 865 – 876 . 10.1016/J.TINS.2022.08.006 36089406

[b8] Caverzasi , E. , Papinutto , N. , Castellano , A. , Zhu , A. H. , Scifo , P. , Riva , M. , Bello , L. , Falini , A. , Bharatha , A. , & Henry , R. G. ( 2016 ). Neurite orientation dispersion and density imaging color maps to characterize brain diffusion in neurologic disorders . Journal of Neuroimaging: Official Journal of the American Society of Neuroimaging , 26 ( 5 ), 494 – 498 . 10.1111/JON.12359 27214558

[b9] Chamberlain , M. C. ( 2011 ). Bevacizumab for the treatment of recurrent glioblastoma. Clinical medicine insights . Oncology , 5 ( 5 ), 117 . 10.4137/CMO.S7232 21603247 PMC3095028

[b10] Chang , P. D. , Chow , D. S. , Yang , P. H. , Filippi , C. G. , & Lignelli , A. ( 2017 ). Predicting glioblastoma recurrence by early changes in the apparent diffusion coefficient value and signal intensity on FLAIR images . American Journal of Roentgenology , 208 ( 1 ), 57 – 65 . 10.2214/AJR.16.16234 27726412

[b11] Chenevert , T. L. , Stegman , L. D. , Taylor , J. M. G. , Robertson , P. L. , Greenberg , H. S. , Rehemtulla , A. , & Ross , B. D. ( 2000 ). Diffusion magnetic resonance imaging: An early surrogate marker of therapeutic efficacy in brain tumors . Journal of the National Cancer Institute , 92 ( 24 ), 2029 – 2036 . https://academic.oup.com/jnci/article-abstract/92/24/2029/2633587 11121466 10.1093/jnci/92.24.2029

[b12] Chong , S. T. , Liu , X. , Kao , H. W. , Lin , C. Y. E. , Hsu , C. C. H. , Kung , Y. C. , Kuo , K. T. , Huang , C. C. , Lo , C. Y. Z. , Li , Y. , Zhao , G. , & Lin , C. P. ( 2021 ). Exploring peritumoral neural tracts by using neurite orientation dispersion and density imaging . Frontiers in Neuroscience , 15 , 702353 . 10.3389/FNINS.2021.702353 34646116 PMC8502884

[b13] Churchill , N. W. , Caverzasi , E. , Graham , S. J. , Hutchison , M. G. , & Schweizer , T. A. ( 2019 ). White matter during concussion recovery: Comparing diffusion tensor imaging (DTI) and neurite orientation dispersion and density imaging (NODDI) . Human Brain Mapping , 40 ( 6 ), 1908 . 10.1002/HBM.24500 30585674 PMC6865569

[b14] Claes , A. , Idema , A. J. , & Wesseling , P. ( 2007 ). Diffuse glioma growth: A guerilla war . Acta Neuropathologica , 114 ( 5 ), 443 – 458 . 10.1007/S00401-007-0293-7 17805551 PMC2039798

[b15] Colgan , N. , Siow , B. , O’Callaghan , J. M. , Harrison , I. F. , Wells , J. A. , Holmes , H. E. , Ismail , O. , Richardson , S. , Alexander , D. C. , Collins , E. C. , Fisher , E. M. , Johnson , R. , Schwarz , A. J. , Ahmed , Z. , O’Neill , M. J. , Murray , T. K. , Zhang , H. , & Lythgoe , M. F. ( 2016 ). Application of neurite orientation dispersion and density imaging (NODDI) to a tau pathology model of Alzheimer’s disease . NeuroImage , 125 , 739 – 744 . 10.1016/J.NEUROIMAGE.2015.10.043 26505297 PMC4692518

[b16] Cox , S. R. , Ritchie , S. J. , Tucker-Drob , E. M. , Liewald , D. C. , Hagenaars , S. P. , Davies , G. , Wardlaw , J. M. , Gale , C. R. , Bastin , M. E. , & Deary , I. J. ( 2016 ). Ageing and brain white matter structure in 3,513 UK Biobank participants . Nature Communications , 7 ( 1 ), 1 – 13 . 10.1038/ncomms13629 PMC5172385 27976682

[b17] Cuddapah , V. A. , Robel , S. , Watkins , S. , & Sontheimer , H. ( 2014 ). A neurocentric perspective on glioma invasion . Nature Reviews. Neuroscience , 15 ( 7 ), 455 – 465 . 10.1038/NRN3765 24946761 PMC5304245

[b18] De Bonis , P. , Anile , C. , Pompucci , A. , Fiorentino , A. , Balducci , M. , Chiesa , S. , Lauriola , L. , Maira , G. , & Mangiola , A. ( 2013 ). The influence of surgery on recurrence pattern of glioblastoma . Clinical Neurology and Neurosurgery , 115 ( 1 ), 37 – 43 . 10.1016/J.CLINEURO.2012.04.005 22537870

[b19] De Leeuw , C. N. , & Vogelbaum , M. A. ( 2019 ). Supratotal resection in glioma: A systematic review . Neuro-Oncology , 21 ( 2 ), 179 – 188 . 10.1093/NEUONC/NOY166 30321384 PMC6374756

[b20] Figini , M. , Riva , M. , Graham , M. , Castelli , G. , Fernandes , B. , Grimaldi , M. , Baselli , G. , Pessina , F. , Bello , L. , Zhang , H. , & Bizzi , A. ( 2018 ). Prediction of isocitrate dehydrogenase genotype in brain gliomas with MRI: Single-shell versus multishell diffusion models . Radiology , 289 . 10.1148/radiol.2018180054 30277427

[b21] Fu , X. , Shrestha , S. , Sun , M. , Wu , Q. , Luo , Y. , Zhang , X. , Yin , J. , & Ni , H. ( 2020 ). Microstructural white matter alterations in mild cognitive impairment and Alzheimer’s disease: Study based on neurite orientation dispersion and density imaging (NODDI) . Clinical Neuroradiology , 30 ( 3 ), 569 – 579 . 10.1007/S00062-019-00805-0 31175374

[b22] Gong , T. , Tong , Q. , He , H. , Sun , Y. , Zhong , J. , & Zhang , H. ( 2020 ). MTE-NODDI: Multi-TE NODDI for disentangling non-T2-weighted signal fractions from compartment-specific T2 relaxation times . NeuroImage , 217 , 116906 . 10.1016/J.NEUROIMAGE.2020.116906 32387626

[b23] Hadjipanayis , C. G. , Widhalm , G. , & Stummer , W. ( 2015 ). What is the surgical benefit of utilizing 5-aminolevulinic acid for fluorescence-guided surgery of malignant gliomas? Neurosurgery , 77 ( 5 ), 663 – 673 . 10.1227/NEU.0000000000000929 26308630 PMC4615466

[b24] Jackson , C. , Choi , J. , Khalafallah , A. M. , Price , C. , Bettegowda , C. , Lim , M. , Gallia , G. , Weingart , J. , Brem , H. , & Mukherjee , D. ( 2020 ). A systematic review and meta-analysis of supratotal versus gross total resection for glioblastoma . Journal of Neuro-Oncology , 148 ( 3 ), 419 – 431 . 10.1007/S11060-020-03556-Y 32562247

[b25] Jin , Y. , Randall , J. W. , Elhalawani , H. , Al Feghali , K. A. , Elliott , A. M. , Anderson , B. M. , Lacerda , L. , Tran , B. L. , Mohamed , A. S. , Brock , K. K. , Fuller , C. D. , & Chung , C. ( 2020 ). Detection of glioblastoma subclinical recurrence using serial diffusion tensor imaging . Cancers , 12 ( 3 ), 568 . 10.3390/CANCERS12030568 32121471 PMC7139975

[b26] Kadota , Y. , Hirai , T. , Azuma , M. , Hattori , Y. , Khant , Z. A. , Hori , M. , Saito , K. , Yokogami , K. , & Takeshima , H. ( 2020 ). Differentiation between glioblastoma and solitary brain metastasis using neurite orientation dispersion and density imaging . Journal of Neuroradiology , 47 ( 3 ), 197 – 202 . 10.1016/J.NEURAD.2018.10.005 30439396

[b27] Kamagata , K. , Zalesky , A. , Hatano , T. , Ueda , R. , Di Biase , M. A. , Okuzumi , A. , Shimoji , K. , Hori , M. , Caeyenberghs , K. , Pantelis , C. , Hattori , N. , & Aoki , S. ( 2017 ). Gray matter abnormalities in idiopathic Parkinson’s disease: Evaluation by diffusional kurtosis imaging and neurite orientation dispersion and density imaging . Human Brain Mapping , 38 ( 7 ), 3704 – 3722 . 10.1002/HBM.23628 28470878 PMC6867088

[b28] Kamiya , K. , Hori , M. , & Aoki , S. ( 2020 ). NODDI in clinical research . Journal of Neuroscience Methods , 346 , 108908 . 10.1016/J.JNEUMETH.2020.108908 32814118

[b29] Kim , M. M. , Sun , Y. , Aryal , M. P. , Parmar , H. A. , Piert , M. , Rosen , B. , Mayo , C. S. , Balter , J. M. , Schipper , M. , Gabel , N. , Briceño , E. M. , You , D. , Heth , J. , Al-Holou , W. , Umemura , Y. , Leung , D. , Junck , L. , Wahl , D. R. , Lawrence , T. S. , & Cao , Y. ( 2021 ). A phase 2 study of dose-intensified chemoradiation using biologically based target volume definition in patients with newly diagnosed glioblastoma . International Journal of Radiation Oncology, Biology, Physics , 110 ( 3 ), 792 – 803 . 10.1016/j.ijrobp.2021.01.033 33524546 PMC8920120

[b30] Kono , K. , Inoue , Y. , Nakayama , K. , Shakudo , M. , Morino , M. , Ohata , K. , Wakasa , K. , & Yamada , R. ( 2001 ). The role of diffusion-weighted imaging in patients with brain tumors . AJNR: American Journal of Neuroradiology , 22 ( 6 ), 1081 . 10.18535/jmscr/v6i2.95 11415902 PMC7974804

[b31] Kraguljac , N. V. , Monroe , W. S. , Anthony , T. , Jindal , R. D. , Hill , H. , & Lahti , A. C. ( 2021 ). Neurite orientation dispersion and density imaging (NODDI) and duration of untreated psychosis in antipsychotic medication-naïve first episode psychosis patients . Neuroimage: Reports , 1 ( 1 ), 100005 . 10.1016/J.YNIRP.2021.100005 36969709 PMC10038586

[b32] Kubben , P. L. , ter Meulen , K. J. , Schijns , O. E. M. G. , ter Laak-Poort , M. P. , van Overbeeke , J. J. , & van Santbrink , H. ( 2011 ). Intraoperative MRI-guided resection of glioblastoma multiforme: A systematic review . The Lancet Oncology , 12 ( 11 ), 1062 – 1070 . 10.1016/S1470-2045(11)70130-9 21868286

[b33] Laack , N. N. , Pafundi , D. , Anderson , S. K. , Kaufmann , T. , Lowe , V. , Hunt , C. , Vogen , D. , Yan , E. , Sarkaria , J. , Brown , P. , Kizilbash , S. , Uhm , J. , Ruff , M. , Zakhary , M. , Zhang , Y. , Seaberg , M. , Wan Chan Tseung , H. S. , Kabat , B. , Kemp , B. , & Brinkmann , D. ( 2021 ). Initial results of a phase 2 trial of 18F-DOPA PET-guided dose-escalated radiation therapy for glioblastoma . International Journal of Radiation Oncology Biology Physics , 110 ( 5 ), 1383 – 1395 . 10.1016/j.ijrobp.2021.03.032 33771703

[b34] Lampinen , B. , Szczepankiewicz , F. , Novén , M. , van Westen , D. , Hansson , O. , Englund , E. , Mårtensson , J. , Westin , C. F. , & Nilsson , M. ( 2019 ). Searching for the neurite density with diffusion MRI: Challenges for biophysical modeling . Human Brain Mapping , 40 ( 8 ), 2529 – 2545 . 10.1002/HBM.24542 30802367 PMC6503974

[b35] Lasocki , A. , & Gaillard , F. ( 2019 ). Non-contrast-enhancing tumor: A new frontier in glioblastoma research . AJNR: American Journal of Neuroradiology , 40 ( 5 ), 758 . 10.3174/AJNR.A6025 30948373 PMC7053910

[b36] Lathia , J. D. , Heddleston , J. M. , Venere , M. , & Rich , J. N. ( 2011 ). Cell stem cell minireview deadly teamwork: Neural cancer stem cells and the tumor microenvironment . Stem Cell , 8 , 482 – 485 . 10.1016/j.stem.2011.04.013 PMC3494093 21549324

[b37] Le Bihan , D. ( 2014 ). Diffusion MRI: What water tells us about the brain . EMBO Molecular Medicine , 6 ( 5 ), 569 – 573 . 10.1002/EMMM.201404055 24705876 PMC4023879

[b81] Laprie , A. , Noel , G. , Chaltiel , L. , Truc , G. , Sunyach , M.-P. , Charissoux , M. , Magne , N. , Auberdiac , P. , Biau , J. , Ken , S. , Tensaouti , F. , Khalifa , J. , Sidibe , I. , Roux , F.-E. , Vieillevigne , L. , Catalaa , I. , Boetto , S. , Uro-Coste , E. , Supiot , S. ,… Cohen-Jonathan-Moyal , E. ( 2024 ). Randomized phase III trial of metabolic imaging-guided dose escalation of radio-chemotherapy in patients with newly diagnosed glioblastoma (SPECTRO GLIO trial) . Neuro-Oncology , 26 ( 1 ), 153 – 163 . 10.1093/neuonc/noad119 37417948 PMC10768994

[b38] Lemercier , P. , Maya , S. P. , Patrie , J. T. , Flors , L. , & Leiva-Salinas , C. ( 2014 ). Gradient of apparent diffusion coefficient values in peritumoral edema helps in differentiation of glioblastoma from solitary metastatic lesions . American Journal of Roentgenology , 203 ( 1 ), 163 – 169 . 10.2214/AJR.13.11186 24951211

[b39] Maier , S. E. , Sun , Y. , & Mulkern , R. V. ( 2010 ). Diffusion imaging of brain tumors . NMR in Biomedicine , 23 ( 7 ), 849 . 10.1002/NBM.1544 20886568 PMC3000221

[b40] Mao , J. , Zeng , W. , Zhang , Q. , Yang , Z. , Yan , X. , Zhang , H. , Wang , M. , Yang , G. , Zhou , M. , & Shen , J. ( 2020 ). Differentiation between high-grade gliomas and solitary brain metastases: A comparison of five diffusion-weighted MRI models . BMC Medical Imaging , 20 ( 1 ), 1 – 11 . 10.1186/S12880-020-00524-W PMC7684933 33228564

[b41] Masjoodi , S. , Hashemi , H. , Oghabian , M. A. , & Sharifi , G. ( 2018 ). Differentiation of edematous, tumoral and normal areas of brain using diffusion tensor and neurite orientation dispersion and density imaging . Journal of Biomedical Physics & Engineering , 8 ( 3 ), 251 . 10.31661/jbpe.v0i0.874 30320029 PMC6169116

[b42] Mastropietro , A. , Rizzo , G. , Fontana , L. , Figini , M. , Bernardini , B. , Straffi , L. , Marcheselli , S. , Ghirmai , S. , Nuzzi , N. P. , Malosio , M. L. , & Grimaldi , M. ( 2019 ). Microstructural characterization of corticospinal tract in subacute and chronic stroke patients with distal lesions by means of advanced diffusion MRI . Neuroradiology , 61 ( 9 ), 1033 . 10.1007/S00234-019-02249-2 31263922 PMC6689031

[b43] Maximov , I. I. , Tonoyan , A. S. , & Pronin , I. N. ( 2017 ). Differentiation of glioma malignancy grade using diffusion MRI . Physica Medica: PM: An International Journal Devoted to the Applications of Physics to Medicine and Biology: Official Journal of the Italian Association of Biomedical Physics (AIFB) , 40 , 24 – 32 . 10.1016/J.EJMP.2017.07.002 28712716

[b44] Miller , K. D. , Ostrom , Q. T. , Kruchko , C. , Patil , N. , Tihan , T. , Cioffi , G. , Fuchs , H. E. , Waite , K. A. , Jemal , A. , Siegel , R. L. , & Barnholtz-Sloan , J. S. ( 2021 ). Brain and other central nervous system tumor statistics, 2021 . CA: A Cancer Journal for Clinicians , 71 ( 5 ), 381 – 406 . 10.3322/CAAC.21693 34427324

[b45] Mitchell , T. , Archer , D. B. , Chu , W. T. , Coombes , S. A. , Lai , S. , Wilkes , B. J. , McFarland , N. R. , Okun , M. S. , Black , M. L. , Herschel , E. , Simuni , T. , Comella , C. , Xie , T. , Li , H. , Parrish , T. B. , Kurani , A. S. , Corcos , D. M. , & Vaillancourt , D. E. ( 2019 ). Neurite orientation dispersion and density imaging (NODDI) and free-water imaging in Parkinsonism . Human Brain Mapping , 40 ( 17 ), 5094 – 5107 . 10.1002/HBM.24760 31403737 PMC6865390

[b46] Nazeri , A. , Mulsant , B. H. , Rajji , T. K. , Levesque , M. L. , Pipitone , J. , Stefanik , L. , Shahab , S. , Roostaei , T. , Wheeler , A. L. , Chavez , S. , & Voineskos , A. N. ( 2017 ). Gray matter neuritic microstructure deficits in schizophrenia and bipolar disorder . Biological Psychiatry , 82 ( 10 ), 726 – 736 . 10.1016/J.BIOPSYCH.2016.12.005 28073491

[b47] Niyazi , M. , Andratschke , N. , Bendszus , M. , Chalmers , A. J. , Erridge , S. C. , Galldiks , N. , Lagerwaard , F. J. , Navarria , P. , Munck af Rosenschöld , P. , Ricardi , U. , van den Bent , M. J. , Weller , M. , Belka , C. , & Minniti , G. ( 2023 ). ESTRO-EANO guideline on target delineation and radiotherapy details for glioblastoma . Radiotherapy and Oncology , 184 , 109663 . 10.1016/J.RADONC.2023.109663 37059335

[b48] Okita , Y. , Takano , K. , Tateishi , S. , Hayashi , M. , Sakai , M. , Kinoshita , M. , Kishima , H. , & Nakanishi , K. ( 2023 ). Neurite orientation dispersion and density imaging and diffusion tensor imaging to facilitate distinction between infiltrating tumors and edemas in glioblastoma . Magnetic Resonance Imaging , 100 , 18 – 25 . 10.1016/J.MRI.2023.03.001 36924806

[b49] Omuro , A. , & DeAngelis , L. M. ( 2013 ). Glioblastoma and other malignant gliomas: A clinical review . JAMA , 310 ( 17 ), 1842 – 1850 . 10.1001/JAMA.2013.280319 24193082

[b50] Onishi , R. , Sawaya , R. , Tsuji , K. , Arihara , N. , Ohki , A. , Ueda , J. , Hata , J. , & Saito , S. ( 2022 ). Evaluation of temozolomide treatment for glioblastoma using amide proton transfer imaging and diffusion MRI . Cancers , 14 ( 8 ), 1907 . 10.3390/CANCERS14081907 35454814 PMC9031574

[b51] Palacios , E. M. , Owen , J. P. , Yuh , E. L. , Wang , M. B. , Vassar , M. J. , Ferguson , A. R. , Diaz-Arrastia , R. , Giacino , J. T. , Okonkwo , D. O. , Robertson , C. S. , Stein , M. B. , Temkin , N. , Jain , S. , McCrea , M. , MacDonald , C. L. , Levin , H. S. , Manley , G. T. , & Mukherjee , P. ( 2020 ). The evolution of white matter microstructural changes after mild traumatic brain injury: A longitudinal DTI and NODDI study . Science Advances , 6 ( 32 ), eaaz6892 . 10.1126/SCIADV.AAZ6892 32821816 PMC7413733

[b52] Parker , T. D. , Slattery , C. F. , Zhang , J. , Nicholas , J. M. , Paterson , R. W. , Foulkes , A. J. M. , Malone , I. B. , Thomas , D. L. , Modat , M. , Cash , D. M. , Crutch , S. J. , Alexander , D. C. , Ourselin , S. , Fox , N. C. , Zhang , H. , & Schott , J. M. ( 2018 ). Cortical microstructure in young onset Alzheimer’s disease using neurite orientation dispersion and density imaging . Human Brain Mapping , 39 ( 7 ), 3005 – 3017 . 10.1002/HBM.24056 29575324 PMC6055830

[b53] Petrecca , K. , Guiot , M. C. , Panet-Raymond , V. , & Souhami , L. ( 2013 ). Failure pattern following complete resection plus radiotherapy and temozolomide is at the resection margin in patients with glioblastoma . Journal of Neuro-Oncology , 111 ( 1 ), 19 – 23 . 10.1007/S11060-012-0983-4 23054563

[b54] Pollard , S. M. , Yoshikawa , K. , Clarke , I. D. , Danovi , D. , Stricker , S. , Russell , R. , Bayani , J. , Head , R. , Lee , M. , Bernstein , M. , Squire , J. A. , Smith , A. , & Dirks , P. ( 2009 ). Glioma stem cell lines expanded in adherent culture have tumor-specific phenotypes and are suitable for chemical and genetic screens . Cell Stem Cell , 4 ( 6 ), 568 – 580 . 10.1016/J.STEM.2009.03.014 19497285

[b55] Price , S. J. , & Gillard , J. H. ( 2011 ). Imaging biomarkers of brain tumour margin and tumour invasion . The British Journal of Radiology , 84 ( Spec Iss 2 ), S159 . 10.1259/BJR/26838774 22433826 PMC3473903

[b56] Price , S. J. , Jena , R. , Burnet , N. G. , Hutchinson , P. J. , Dean , A. F. , Peña , A. , Pickard , J. D. , Carpenter , T. A. , & Gillard , J. H. ( 2006 ). Improved delineation of glioma margins and regions of infiltration with the use of diffusion tensor imaging: An image-guided biopsy study . American Journal of Neuroradiology , 27 ( 9 ), 1969 – 1974 . 10.1016/s0513-5117(08)79144-0 17032877 PMC7977915

[b82] Ramesh , K. , Mellon , E. A. , Gurbani , S. S. , Weinberg , B. D. , Schreibmann , E. , Sheriff , S. A. , Goryawala , M. , De Le Fuente , M. , Eaton , B. R. , Zhong , J. , Voloschin , A. D. , Sengupta , S. , Dunbar , E. M. , Holdhoff , M. , Barker , P. B. , Maudsley , A. A. , Kleinberg , L. R. , Shim , H. , & Shu , H. K. G. ( 2022 ). A multi-institutional pilot clinical trial of spectroscopic MRI-guided radiation dose escalation for newly diagnosed glioblastoma . Neuro-Oncology Advances , 4 ( 1 ), vdac006. 10.1093/noajnl/vdac006 PMC8976280 35382436

[b57] Sadeghi , N. , D’Haene , N. , Decaestecker , C. , Levivier , M. , Metens , T. , Maris , C. , Wikler , D. , Baleriaux , D. , Salmon , I. , & Goldman , S. ( 2008 ). Apparent diffusion coefficient and cerebral blood volume in brain gliomas: Relation to tumor cell density and tumor microvessel density based on stereotactic biopsies . American Journal of Neuroradiology , 29 ( 3 ), 476 – 482 . 10.3174/AJNR.A0851 18079184 PMC8118877

[b58] Sanvito , F. , Castellano , A. , & Falini , A. ( 2021 ). Advancements in neuroimaging to unravel biological and molecular features of brain tumors . Cancers , 13 ( 3 ), 424 – 424 . 10.3390/CANCERS13030424 33498680 PMC7865835

[b59] Schatlo , B. , Fandino , J. , Smoll , N. R. , Wetzel , O. , Remonda , L. , Marbacher , S. , Perrig , W. , Landolt , H. , & Fathi , A. R. ( 2015 ). Outcomes after combined use of intraoperative MRI and 5-aminolevulinic acid in high-grade glioma surgery . Neuro-Oncology , 17 ( 12 ), 1560 – 1567 . 10.1093/NEUONC/NOV049 25858636 PMC4633924

[b60] Schneider , T. , Brownlee , W. , Zhang , H. , Ciccarelli , O. , Miller , D. H. , & Wheeler-Kingshott , C. G. ( 2017 ). Sensitivity of multi-shell NODDI to multiple sclerosis white matter changes: A pilot study . Functional Neurology , 32 ( 2 ), 97 – 101 . 10.11138/FNEUR/2017.32.2.097 28676143 PMC5507159

[b61] Seano , G. , Nia , H. T. , Emblem , K. E. , Datta , M. , Ren , J. , Krishnan , S. , Kloepper , J. , Pinho , M. C. , Ho , W. W. , Ghosh , M. , Askoxylakis , V. , Ferraro , G. B. , Riedemann , L. , Gerstner , E. R. , Batchelor , T. T. , Wen , P. Y. , Lin , N. U. , Grodzinsky , A. J. , Fukumura , D. , … Jain , R. K. ( 2019 ). Solid stress in brain tumours causes neuronal loss and neurological dysfunction and can be reversed by lithium . Nature Biomedical Engineering , 3 ( 3 ), 230 – 245 . 10.1038/s41551-018-0334-7 PMC6452896 30948807

[b62] Sone , D. , Sato , N. , Ota , M. , Maikusa , N. , Kimura , Y. , & Matsuda , H. ( 2018 ). Abnormal neurite density and orientation dispersion in unilateral temporal lobe epilepsy detected by advanced diffusion imaging . NeuroImage. Clinical , 20 , 772 – 782 . 10.1016/J.NICL.2018.09.017 30268026 PMC6169249

[b63] Spano , B. , Giulietti , G. , Pisani , V. , Morreale , M. , Tuzzi , E. , Nocentini , U. , Francia , A. , Caltagirone , C. , Bozzali , M. , & Cercignani , M. ( 2018 ). Disruption of neurite morphology parallels MS progression . Neurology-Neuroimmunology Neuroinflammation , 5 ( 6 ), 502 . 10.1212/NXI.0000000000000502 PMC6192688 30345330

[b64] Stepp , H. , & Stummer , W. ( 2018 ). 5-ALA in the management of malignant glioma . Lasers in Surgery and Medicine , 50 ( 5 ), 399 – 419 . 10.1002/LSM.22933 29737540

[b65] Stummer , W. , Pichlmeier , U. , Meinel , T. , Wiestler , O. D. , Zanella , F. , & Reulen , H. J. ( 2006 ). Fluorescence-guided surgery with 5-aminolevulinic acid for resection of malignant glioma: A randomised controlled multicentre phase III trial . The Lancet Oncology , 7 ( 5 ), 392 – 401 . 10.1016/S1470-2045(06)70665-9 16648043

[b66] Stupp , R. , Mason , W. P. , van den Bent , M. J. , Weller , M. , Fisher , B. , Taphoorn , M. J. B. , Belanger , K. , Brandes , A. A. , Marosi , C. , Bogdahn , U. , Curschmann , J. , Janzer , R. C. , Ludwin , S. K. , Gorlia , T. , Allgeier , A. , Lacombe , D. , Cairncross , J. G. , Eisenhauer , E. , & Mirimanoff , R. O. ( 2005 ). Radiotherapy plus concomitant and adjuvant temozolomide for glioblastoma . New England Journal of Medicine , 352 ( 10 ), 987 – 996 . 10.1056/NEJMOA043330 15758009

[b67] Sugahara , T. , Korogi , Y. , Kochi , M. , Ikushima , I. , Shigematu , Y. , Hirai , T. , Okuda , T. , Liang , L. , Ge , Y. , Komohara , Y. , Ushio , Y. , & Takahashi , M. ( 1999 ). Usefulness of diffusion-weighted MRI with echo-planar technique in the evaluation of cellularity in gliomas . Journal of Magnetic Resonance Imaging , 9 ( 1 ), 53 – 60 . 10.1002/(sici)1522-2586(199901)9:1<53::aid-jmri7>3.0.co;2-2 10030650

[b68] Thoeny , H. C. , & Ross , B. D. ( 2010 ). Predicting and monitoring cancer treatment response with diffusion-weighted MRI . Journal of Magnetic Resonance Imaging , 32 ( 1 ), 2 – 16 . 10.1002/JMRI.22167 20575076 PMC2918419

[b69] Vehlow , A. , & Cordes , N. ( 2013 ). Invasion as target for therapy of glioblastoma multiforme . Biochimica et Biophysica Acta (BBA)-Reviews on Cancer , 1836 ( 2 ), 236 – 244 . 10.1016/J.BBCAN.2013.07.001 23891970

[b70] Venkataramani , V. , Tanev , D. I. , Strahle , C. , Studier-Fischer , A. , Fankhauser , L. , Kessler , T. , Körber , C. , Kardorff , M. , Ratliff , M. , Xie , R. , Horstmann , H. , Messer , M. , Paik , S. P. , Knabbe , J. , Sahm , F. , Kurz , F. T. , Acikgöz , A. A. , Herrmannsdörfer , F. , Agarwal , A. , … Kuner , T. ( 2019 ). Glutamatergic synaptic input to glioma cells drives brain tumour progression . Nature , 573 ( 7775 ), 532 – 538 . 10.1038/S41586-019-1564-X 31534219

[b71] Veraart , J. , Fieremans , E. , & Novikov , D. S. ( 2016 ). Diffusion MRI noise mapping using random matrix theory . Magnetic Resonance in Medicine , 76 ( 5 ), 1582 – 1593 . 10.1002/MRM.26059 26599599 PMC4879661

[b72] Wang , J. , Xu , S. L. , Duan , J. J. , Yi , L. , Guo , Y. F. , Shi , Y. , Li , L. , Yang , Z. Y. , Liao , X. M. , Cai , J. , Zhang , Y. Q. , Xiao , H. L. , Yin , L. , Wu , H. , Zhang , J. N. , Lv , S. Q. , Yang , Q. K. , Yang , X. J. , Jiang , T. , … Yu , S. C. ( 2018 ). Invasion of white matter tracts by glioma stem cells is regulated by a NOTCH1–SOX2 positive-feedback loop . Nature Neuroscience , 22 ( 1 ), 91 – 105 . 10.1038/s41593-018-0285-z 30559479

[b73] Wang , Z. , Zhang , S. , Liu , C. , Yao , Y. , Shi , J. , Zhang , J. , Qin , Y. , & Zhu , W. ( 2019 ). A study of neurite orientation dispersion and density imaging in ischemic stroke . Magnetic Resonance Imaging , 57 , 28 – 33 . 10.1016/J.MRI.2018.10.018 30385381

[b74] Wen , Q. , Kelley , D. A. C. , Banerjee , S. , Lupo , J. M. , Chang , S. M. , Xu , D. , Hess , C. P. , & Nelson , S. J. ( 2015 ). Clinically feasible NODDI characterization of glioma using multiband EPI at 7 T . NeuroImage: Clinical , 9 , 291 – 299 . 10.1016/J.NICL.2015.08.017 26509116 PMC4579286

[b75] Winston , G. P. , Micallef , C. , Symms , M. R. , Alexander , D. C. , Duncan , J. S. , & Zhang , H. ( 2014 ). Advanced diffusion imaging sequences could aid assessing patients with focal cortical dysplasia and epilepsy . Epilepsy Research , 108 ( 2 ), 336 – 339 . 10.1016/J.EPLEPSYRES.2013.11.004 24315018 PMC3969285

[b76] Yi , S. Y. , Barnett , B. R. , Torres-Velázquez , M. , Zhang , Y. , Hurley , S. A. , Rowley , P. A. , Hernando , D. , & Yu , J. P. J. ( 2019 ). Detecting microglial density with quantitative multi-compartment diffusion MRI . Frontiers in Neuroscience , 13 , 81 . 10.3389/FNINS.2019.00081 30837826 PMC6389825

[b77] Zhang , H. , Schneider , T. , Wheeler-Kingshott , C. A. , & Alexander , D. C. ( 2012 ). NODDI: Practical in vivo neurite orientation dispersion and density imaging of the human brain . NeuroImage , 61 ( 4 ), 1000 – 1016 . 10.1016/J.NEUROIMAGE.2012.03.072 22484410

[b78] Zhao , J. , Li , J. B. , Wang , Y, J. , Wang , Y. L. , Liu , D. W. , Li , X. B. , Song , Y. K. , Tian , Y. S. , Yan , X. , Li , Z. H. , He , S. F. , Huang , X. L. , Jiang , L. , Yang , Z. Y. , & Chu , J. P. ( 2018 ). Quantitative analysis of neurite orientation dispersion and density imaging in grading gliomas and detecting IDH-1 gene mutation status . NeuroImage: Clinical , 19 , 174 . 10.1016/J.NICL.2018.04.011 30023167 PMC6050458

