# Supplementary material for: Detecting glioblastoma infiltration beyond conventional imaging tumour margins using MTE-NODDI
Source: Imaging Neurosci (Camb). 2025 Feb 18;3:imag_a_00472. doi: 10.1162/imag_a_00472 (PMC12319949; doi:10.1162/imag_a_00472)
Supplement: Supplementary Material [file imag_a_00472-supp.pdf]

Appendix. Supplementary data

Supplementary Figure 1.

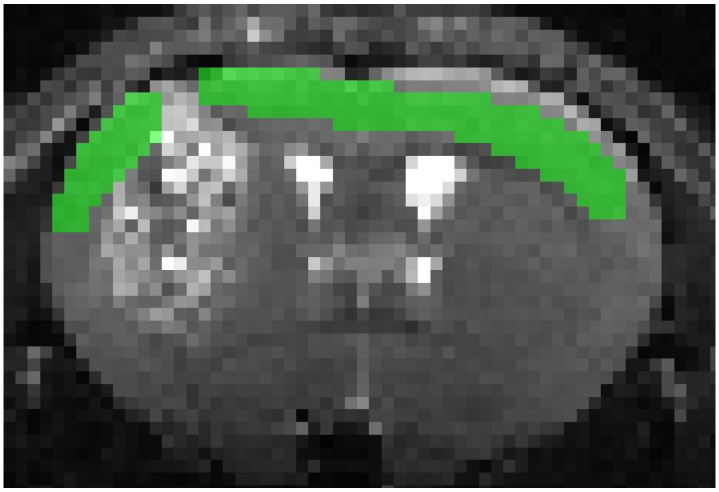

Representative coronal T2WI image with cortical ROI for histology correlation. For illustrative purposes, cortical ROI is shown in green.

Supplementary Figure 2.

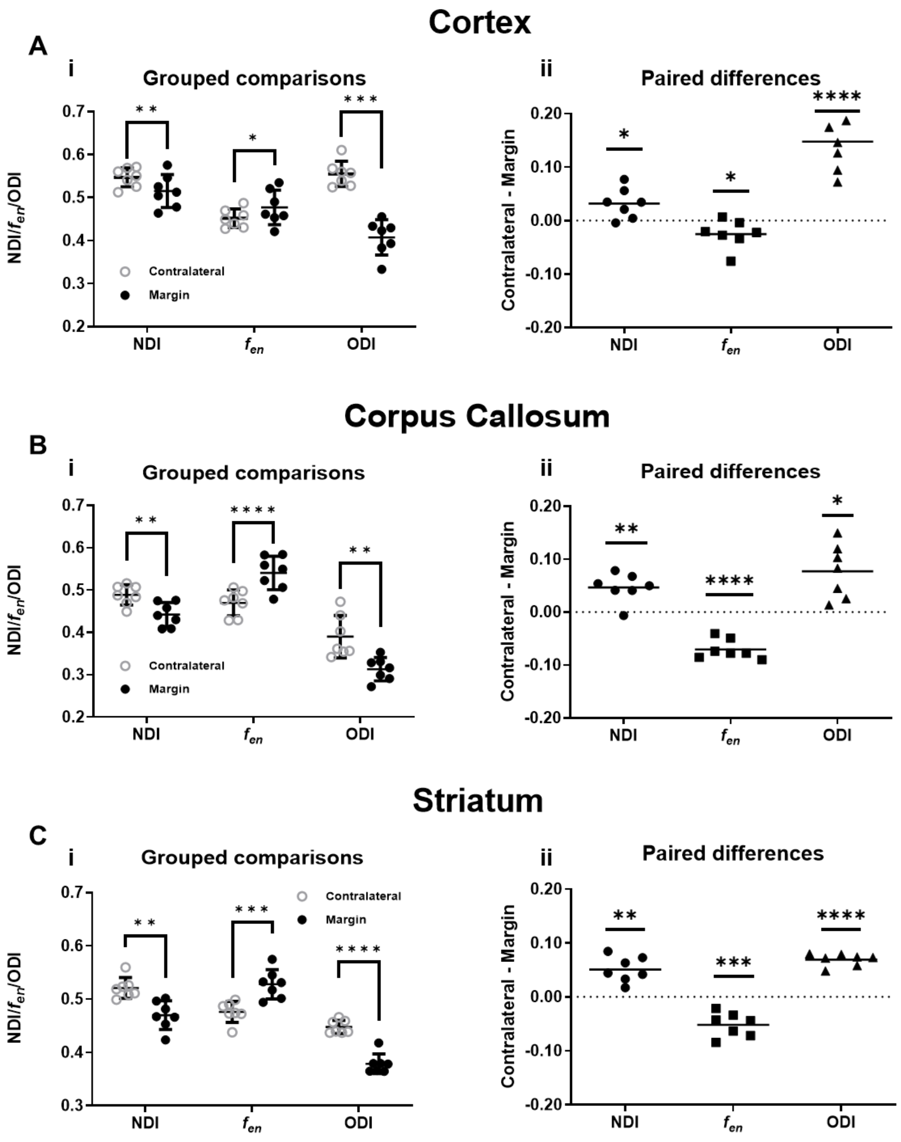

MTE-NODDI parameters from the peritumoural margin compared to contralateral regions for each animal at the 8-week time point. NDI, f_{en}, and ODI values from the contralateral and peritumoural margins in **(A)** Cortex, **(B)** Corpus callosum, and **(C)** Striatum. Within each panel: (i) displays grouped comparisons between contralateral and peritumoural margins. Each data point on the plots represents the mean value for individual mouse (n=7). (ii) is a plot of paired differences (contralateral – peritumoural margins) for each mouse (n=7). Paired t-tests were used to test statistically significant differences between contralateral and peritumoural margins. (* = p < 0.05, ** = p < 0.005, *** = p < 0.001, *** = p < 0.0005).

Supplementary Figure 3.

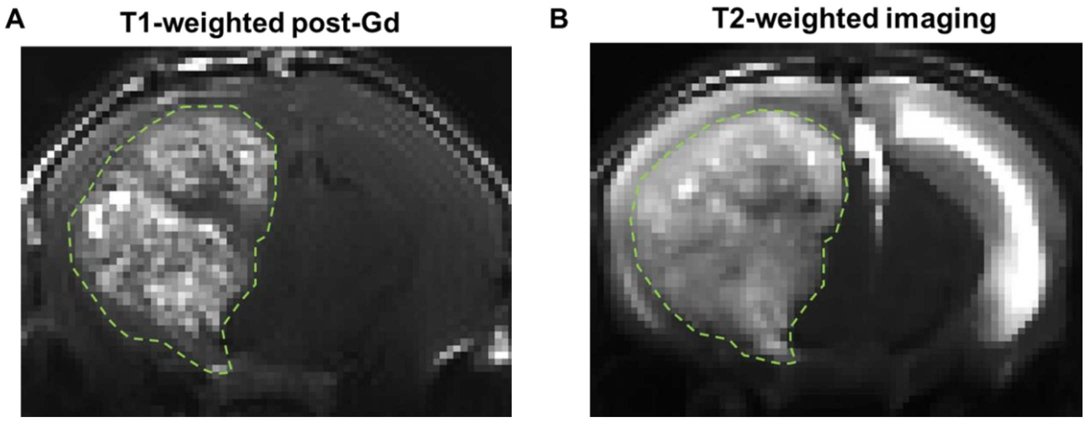

Comparison of tumour volumes measured using T1-weighted post-Gadolinium and T2-weighted imaging (n=2). (A) T1-weighted post-Gadolinium image showing contrast-enhanced tumour bulk. **(B)** T2-weighted sequence used in the current study to define tumour regions of interest. Tumours regions on both modalities are indicated by dashed green line.

Supplementary Figure 4.

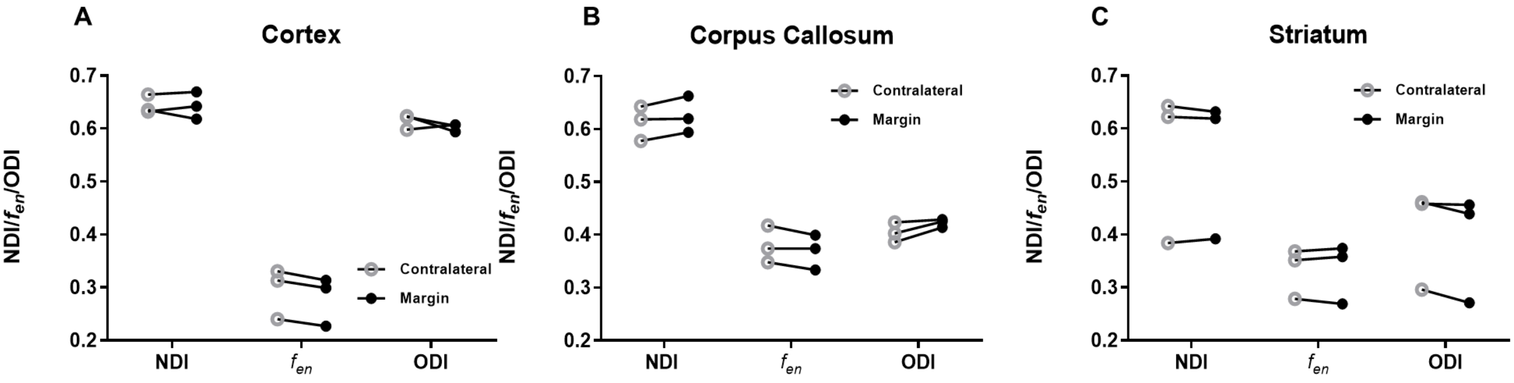

Effect of ROI displacement caused due to tumour mass on measurements of MTE-NODDI parameters (n=3) at the 8-week timepoint. NDI, f_{en}, and ODI values from the contralateral and peritumoural margins in age- and strain-matched naïve mice in **(A)** Cortex, **(B)** Corpus callosum, and **(C)** Striatum. Each data point on the plots represents the mean value for individual mouse. Paired t-tests were used to test statistically significant differences between contralateral and peritumoural margins. No significant differences were observed.

Supplementary Figure 5.

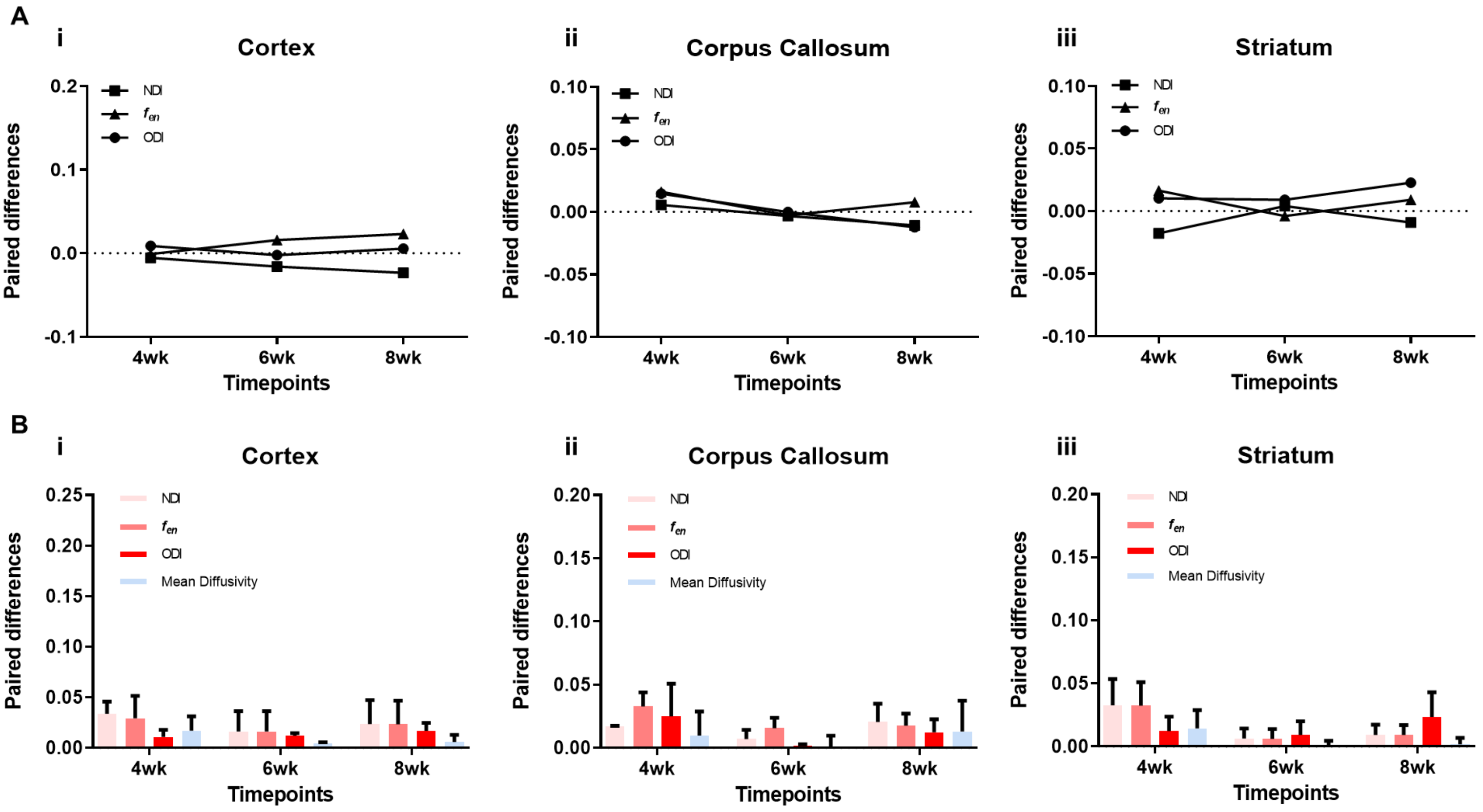

MTE-NODDI and MD parameters at the three imaging timepoints from age-matched and strain-matched naïve mice. (A) Paired differences (Contralateral – Peritumoural Margin) of NDI, f_{en}, and ODI at 4wk, 6wk, and 8wk timepoints in (i) Cortex, (ii) Corpus callosum, and (iii) Striatum. Each data point in the plot represents the mean of paired differences from all animals (n=3). **(B)** Paired differences (Contralateral – Peritumoural Margin) of NDI, f_{en}, ODI, and MD at 4wk, 6wk, and 8wk timepoints in (i) Cortex, (ii) Corpus callosum, and (iii) Striatum. Barplots represent means of paired differences from all animals (n=3) where MTE-NODDI parameters are shown in red, and DTI MD is plotted in blue for illustrative purposes. Paired t-tests were used to test statistically significant differences between contralateral and peritumoural margins. No significant differences were observed.

Supplementary Figure 6.

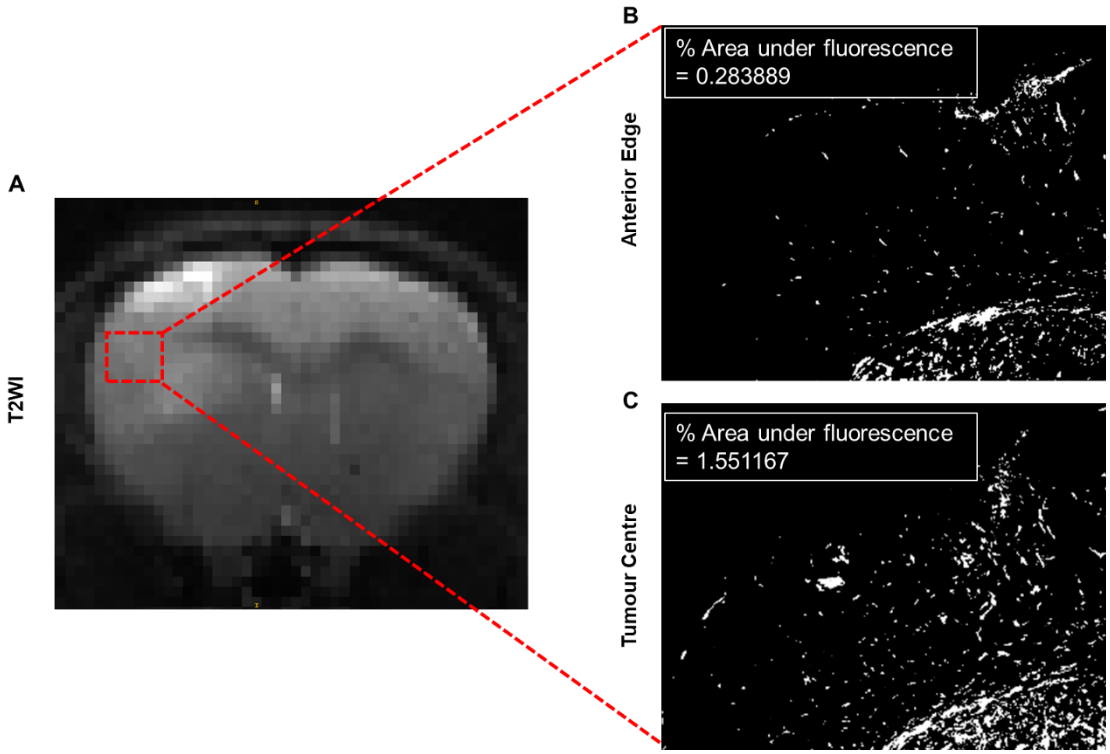

Correlation of MRI parameters to GFP+ fluorescence from additional histological images. Representative T2WI image and GFP+ histological images from a G144 tumour mouse. GFP+ fluorescence calculated as % Area under fluorescence following thresholding on an additional 9 GFP+ slices from the anterior edge of the tumour (B) compared to the center (C) did not show any corelations with the MRI parameters.
